# Stress Hormones Epinephrine and Corticosterone Selectively Reactivate HSV-1 and HSV-2 in Sympathetic and Sensory Neurons

**DOI:** 10.3390/v14051115

**Published:** 2022-05-23

**Authors:** Poorna Goswami, Angela M. Ives, Amber R. N. Abbott, Andrea S. Bertke

**Affiliations:** 1 Translational Biology Medicine and Health, Virginia Polytechnic Institute and State University, Blacksburg, VA 24060, USA; poorna08@vt.edu; 2 Biomedical and Veterinary Science, Virginia-Maryland College of Veterinary Medicine, Virginia Polytechnic Institute and State University, Blacksburg, VA 24060, USA; amives@vt.edu; 3 Department of Biology, Virginia Polytechnic Institute and State University, Blacksburg, VA 24060, USA; ambabbot@vt.edu; 4 Population Health Sciences, Center for Emerging Zoonotic and Arthropod-Borne Pathogens, Virginia-Maryland College of Veterinary Medicine, Virginia Polytechnic Institute and State University, Blacksburg, VA 24060, USA

**Keywords:** herpes simplex virus, HSV-1, HSV-2, epinephrine, corticosterone, reactivation, primary adult neurons, autonomic neurons, sensory neurons

## Abstract

Herpes simplex viruses 1 and 2 (HSV-1 and HSV-2) establish latency in sensory and autonomic neurons, from which they can reactivate to cause recurrent disease throughout the life of the host. Stress is strongly associated with HSV recurrences in humans and animal models. However, the mechanisms through which stress hormones act on the latent virus to cause reactivation are unknown. We show that the stress hormones epinephrine (EPI) and corticosterone (CORT) induce HSV-1 reactivation selectively in sympathetic neurons, but not sensory or parasympathetic neurons. Activation of multiple adrenergic receptors is necessary for EPI-induced HSV-1 reactivation, while CORT requires the glucocorticoid receptor. In contrast, CORT, but not EPI, induces HSV-2 reactivation in both sensory and sympathetic neurons through either glucocorticoid or mineralocorticoid receptors. Reactivation is dependent on different transcription factors for EPI and CORT, and coincides with rapid changes in viral gene expression, although genes differ for HSV-1 and HSV-2, and temporal kinetics differ for EPI and CORT. Thus, stress-induced reactivation mechanisms are neuron-specific, stimulus-specific and virus-specific. These findings have implications for differences in HSV-1 and HSV-2 recurrent disease patterns and frequencies, as well as development of targeted, more effective antivirals that may act on different responses in different types of neurons.

## 1. Introduction

Herpes simplex viruses 1 and 2 (HSV-1 and HSV-2) infect mucosal cells, then establish lifelong latent infection in sensory and autonomic ganglia innervating the site of infection. The viruses can reactivate to cause recurrent disease; HSV-1 is more commonly associated with recurrent orolabial lesions and herpes keratitis, while HSV-2 causes recurrent genital lesions. Although HSV-1 is diagnosed in approximately 30% of new primary genital herpes infections, only 10–25% of individuals infected genitally with HSV-1 experience recurrences, while 60–90% of those infected with HSV-2 experience recurrent genital disease [1,2,3]. Symptomatic and asymptomatic genital herpes recurrences can also be associated with autonomic dysfunction, including urinary retention, constipation, erectile dysfunction, or impotence, although differences between HSV-1 and HSV-2 have not been assessed in human patients [4]. More rarely, HSV-1 causes necrotizing encephalitis with a high mortality rate and HSV-2 causes recurrent sacral meningitis, often unrecognized as an HSV-2-related disease [5,6]. The mechanisms that cause these differences in recurrent disease frequency and anatomical preferences are not understood.

Stress has been defined as a force that elicits a somatic response beyond the regular bodily functions. Any natural stimulus, ranging from physiological and pathological to social and psychological, can cause a stress response under intense pressure [7]. Social, psychological, physical, and persistent stress is strongly correlated with recurrent HSV disease in humans, and the stress hormone epinephrine induces reactivation in animal models [8,9,10,11,12,13]. Receptors for the two major stress hormones, epinephrine and cortisol, are expressed selectively in different types of neurons, including sensory neurons in the trigeminal ganglia (TG) and autonomic neurons in the superior cervical (SCG) and ciliary ganglia (CG) [14,15,16].

Epinephrine (EPI), also known as adrenaline, is a catecholaminergic hormone secreted by the adrenal medulla and regulated by the sympathetic nervous system to induce a “short-term” stress response referred to as the “fight or flight response”. EPI induces HSV-1 reactivation in mouse and rabbit in vivo models of infection [11,12], although adrenergic reactivation is significantly reduced if the latency-associated transcript (LAT) is deleted or mutated [17]. Iontophoresis of EPI during HSV-2 latent infection induces reactivation significantly less frequently compared to HSV-1 in the rabbit eye model [18,19], suggesting that HSV-1 and HSV-2 may be differentially responsive to EPI-induced reactivation. Furthermore, we recently showed that EPI enhances DNA replication and the production of viral progeny during productive infection of HSV-1 in sympathetic but not sensory neurons, demonstrating that EPI acts on HSV preferentially in specific types of neurons [16]. Thus, EPI may selectively induce HSV reactivation in both a neuron- and virus-specific manner.

Cortisol, a glucocorticoid secreted by the adrenal cortex, induces a “long-term” stress response. Cortisol binds to glucocorticoid (GR) and mineralocorticoid receptors (MR), and its major effects on humans and mammals include regulation of metabolism and suppression of the immune system. Cortisol (corticosterone in rodents) induces HSV-1 and HSV-2 reactivation in vivo, which is presumed to occur through immune system suppression [20,21]. However, stress-induced transcription factors expressed in sensory TG neurons stimulate the HSV-1 ICP4, ICP27, and ICP0 promoters [22,23,24,25,26], and ICP0 alone can initiate reactivation of HSV-1 from latency [27,28,29]. In addition, we previously demonstrated that corticosterone (CORT) modulates acute HSV-1 and HSV-2 replication during productive infection in sympathetic but not sensory neurons [16]. Thus, CORT may potentially induce HSV reactivation by acting directly on neurons that harbor latent virus, rather than acting through immune system suppression.

To determine if stress hormones differentially reactivate HSV-1 and HSV-2 at a cellular level in different types of mature neurons, we administered either EPI or CORT to latently infected primary murine adult sensory and autonomic neurons in culture. Quantification of viral replication, production of viral progeny, and gene expression show that stress hormones differentially induce reactivation depending on the type of neuron and the infecting virus. Furthermore, administration of stress hormone receptor agonists and antagonists demonstrate that EPI and CORT exert their effects through specific adrenergic and corticosteroid receptors to selectively induce HSV-1 and HSV-2 reactivation from latency in primary adult sensory and autonomic neurons.

## 2. Materials and Methods

### 2.1. Cell Lines and Virus Strains

HSV-1 strain 17+ was originally isolated and transferred from John Hay (SUNY, Buffalo, NY, USA) to the Krause lab (FDA, Bethesda, MD, USA). HSV-2 strain 333 was originally isolated and transferred from Gary Hayward (Johns Hopkins, Baltimore, MD, USA) to the Krause lab (FDA, Bethesda, MD, USA). Virus was propagated in Vero 76 cells (CRL-1586, ATCC) in the Krause lab and Passage 1 aliquots were stored at −80 °C; most new virus stocks propagated in the Krause lab were propagated from the first passage aliquots, resulting in Passage 2. A Passage 2 aliquot was transferred to the Margolis lab (UCSF, San Francisco, CA, USA); new virus stocks were propagated and stored at −80 °C (Passage 3). A Passage 3 aliquot was transferred to the Bertke lab (Virginia Tech, Blacksburg, VA, USA); new virus stocks were propagated and stored at −80 °C (Passage 4). All virus stocks used in the Bertke lab were propagated from the Passage 4 stocks in Vero 76 cells (Passage 5). Stocks were titrated on Vero 76 cells in quadruplicate to determine concentration. Stock viruses were diluted in Neurobasal A medium (Thermo Fisher, Waltham, MA, USA) for inoculation of primary adult murine neuronal cultures.

### 2.2. Primary Adult Murine Neuronal Cultures

Female six-week-old Swiss Webster mice (Hilltop Laboratories, Scottsdale, PA, USA) were euthanized with CO_2_ and transcardially perfused with cold, calcium- and magnesium-free phosphate-buffered saline (PBS). Sensory trigeminal ganglia (TG), sympathetic superior cervical ganglia (SCG), and parasympathetic ciliary ganglia (CG) were collected into Neurobasal A medium (Gibco) supplemented with penicillin–streptomycin and B27 Supplement (ThermoFisher) on ice. Ganglia were enzymatically dissociated sequentially in papain and collagenase/dispase (Worthington Biochemical, Lakewood, NJ, USA), followed by mechanical trituration by pipette. TG neurons were enriched on Optiprep gradients. SCG and CG did not go through a gradient step because they contain less ganglionic debris after dissociation. Neurons were plated on culture plates coated with Matrigel Matrix Basement Membrane (Corning) and maintained in Complete Neuro Media, consisting of Neurobasal A medium supplemented with B27 Supplement (Thermo Fisher), penicillin–streptomycin (Thermo Fisher), Glutamax (Thermo Fisher), and neurotrophic factors (NTFs from PeproTech, Cranbury, NJ, USA; nerve growth factor (NGF), glial cell-derived neurotrophic factor (GDNF), neurturin (NTN), ciliary neurotrophic factor (CNTF; for CGs only)), and 5-fluorodeoxyuridine (Sigma) to deplete nonneuronal cells [30]. Primary adult neuronal cultures have previously been assessed for maintenance of in vivo characteristics, neuronal markers, and specific receptors relevant to these studies [16,30,31,32]. All animal care and handling were in accordance with the Virginia Tech Institutional Care and Use Committee (IACUC# 15-237 approved 5 February 2016, 18-237 approved 2 August 2019, and 21-244 approved 11 January 2022).

### 2.3. Establishment of Latent Infection

Four days after plating, neurons were inoculated with 30 multiplicity of infection (moi) of HSV-1 (strain 17+) or HSV-2 (strain 333). We previously showed that at 30 moi, 90% of the total neurons become infected and establish latency [30]. After a one-hour adsorption period at 37 °C/5% CO_2_, viral inoculum was removed and replaced with fresh Complete Neuro media with no 5-fluorodeoxyuridine and with 300 µM acyclovir (Sigma, St. Louis, MO, USA) for seven days to establish and maintain a latent infection in vitro.

### 2.4. Reactivation with Stress Hormones

Seven days after establishment of a latent infection, Complete Neuro media with 300 µM acyclovir was removed and replaced with fresh Complete Neuro media with no acyclovir and with either 10 µM epinephrine (Sigma) for adrenergic reactivation or 10 µM corticosterone-HBC (Sigma) for corticosterone-induced reactivation. Four different concentrations of each hormone (0.01, 0.1, 1, 10 µM) were tested for effects during productive infection [16]. To ensure maximum effects, 10 µM epinephrine or corticosterone were used for all reactivation and inhibitor studies. Concentrations for inhibitors were based on previously published characterizations and studies for each agent. Corticosterone-HBC is a water-soluble corticosterone conjugated to (2-Hydroxypropyl)-β-cyclodextrin (HBC), a carrier molecular that enables solubility in media without harming the cultured neurons. HBC was used as a control to ensure that corticosterone and not the vehicle was inducing reactivation. Untreated controls had acyclovir removed but were otherwise untreated to ensure that removal of acyclovir did not promote replication. Approximately 30% of the total neurons reactivate in response to a reactivation stimulus [30,32].

### 2.5. Reactivation with Receptor Agonists & Antagonists

For testing adrenergic receptor specificity, seven days after establishment of a latent infection, media containing acyclovir was removed and Complete Neuro media with no acyclovir and with one, two, or three adrenergic agonists were added. Adrenergic receptor (AR) agonists included phenylephrine (α-1 AR agonist), clonidine (α-2 AR agonist), dobutamine (β-1 AR agonist), or terbutaline (β-2 AR agonist) (Sigma). Adrenergic receptor antagonists phentolamine (nonspecific α-AR antagonist) or timolol (nonspecific β-AR antagonist) were also added with 10 µM epinephrine to fresh Complete Neuro media. For testing corticosterone receptor specificity, Complete Neuro media with no acyclovir and with either the glucocorticoid receptor agonist dexamethasone (GR agonist) or mineralocorticoid receptor agonist aldosterone (MR agonist) (Sigma) were added to Complete Neuro media (10 µM). The corticosterone antagonists mifepristone (GR antagonist), eplerenone (MR antagonist) (Sigma), or both at 10 µM were added to fresh Complete Neuro media with 10 µM corticosterone-HBC.

### 2.6. Reactivation with Protein Inhibitors

For testing molecular inhibition of intermediates in signaling cascades, seven days after establishment of latent infection, media containing acyclovir was removed. Complete Neuro media with no acyclovir and 10 µM β-catenin inhibitor iCRT14 (Sigma), 10 µM CREB inhibitor 666-15 (EMD Millipore), 20 µM JNK inhibitor SP600125 (Sigma), 20 µM PI3-K inhibitor LY294002 (Sigma), 30 μM p300/CBP inhibitor C646 (Sigma), or 1 μM HDAC1 inhibitor pyroxamide (Sigma) with 10 µM epinephrine or corticosterone-HBC were added to primary adult neuronal cultures latently infected with HSV-1 or HSV-2.

### 2.7. Quantitation of HSV Viral Load

Viral DNA was extracted from neuronal cultures 24 h post-reactivation (hpr) with TRI reagent (Thermo Fisher), according to the manufacturer’s instructions. Viral DNA load was determined by qPCR using HSV-1 or HSV-2 thymidine kinase (TK) gene-specific primers and probes. All assays were normalized to 18s rRNA (Applied Biosystems, Waltham, MA, USA) and reported as viral copy number in 200 ng of total DNA, which represents approximately 120–240 neurons.

### 2.8. Plaque Assay

Twenty-four hours post-reactivation, neurons and media were collected and viral titer was determined by standard plaque assay on Vero E6 cells.

### 2.9. Quantitation of HSV Gene Expression

Viral RNA was extracted from neuronal cultures during latency (after 7 days latency, acyclovir was not removed) and between 1 h and 25 h post-reactivation with TRI reagent (Thermo Fisher), according to the manufacturer’s instructions. RNA was reverse-transcribed to cDNA with iScript (Biorad, Hercules, CA, USA). Gene expression was determined by quantifying the HSV-1 or HSV-2 immediate early genes ICP0, ICP4, ICP27, and transactivator VP16 by digital droplet PCR (ddPCR) using HSV-1 or HSV-2 gene-specific primers specific for each gene and EvaGreen QX200 Master mix (Biorad) on a QX200 ddPCR system (BioRad). All assays were normalized to 18s rRNA and reported as transcripts/neuron (calculated from number of neurons per well) and fold-change of epinephrine or corticosterone treated samples compared to untreated neurons (latent for 7 days, acyclovir not removed).

### 2.10. Quantification and Statistical Analysis

All experiments were performed using a minimum of three neuronal cultures performed on different days to ensure reproducibility, with 2–3 biological replicates for each treatment in each culture. All assays were performed in duplicate or triplicate for technical replicates of each sample. Data are presented as means ± standard error of the mean. Statistics were performed using parametric analyses with MS Excel and JMP Pro version 12, including analysis of variance with contrast tests. Where indicated, asterisks denote statistical significance as follows: * *p* < 0.05, ** *p* < 0.01, and *** *p* < 0.001.

## 3. Results

### 3.1. Stress Hormones Differentially Induce HSV Reactivation In Vitro

To determine if stress hormones induce HSV-1 and HSV-2 reactivation from latent infection in different types of mature neurons, primary adult murine sensory trigeminal (TG), sympathetic superior cervical (SCG), and parasympathetic ciliary ganglia (CG) neuronal cultures latently infected with HSV-1 (17 syn+) or HSV-2 (333) were treated with EPI or CORT. We previously determined active concentrations of EPI and CORT that produce biologically and statistically significant effects on productive HSV-1 and HSV-2 infection, without toxic effects in these neurons [16]. Viral DNA was quantified by qPCR with primers and probes specific for the HSV-1 or HSV-2 thymidine kinase (TK) gene.

**Figure 1:**
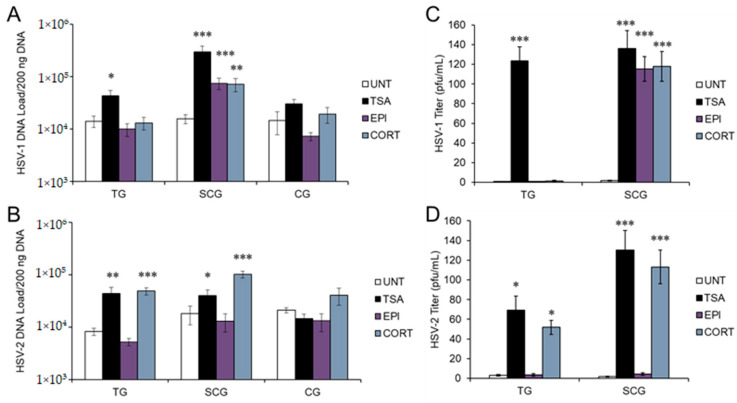
EPI and CORT induce HSV-1 and HSV-2 reactivation selectively in sensory, sympathetic, and parasympathetic neurons. Primary adult neuronal cultures from sensory trigeminal (TG), sympathetic superior cervical (SCG), and parasympathetic ciliary ganglia (CG) were infected with HSV-1 or HSV-2 in the presence of acyclovir for 7 days, followed by treatment with epinephrine (EPI; 10 μM), corticosterone (CORT; 10 μM), or trichostatin A (TSA; 1.2 μM), compared to untreated (UNT) neurons. HSV-1 (**A**) and HSV-2 (**B**) viral DNA was quantified by qPCR 24 h post-reactivation. In parallel, HSV-1 (**C**) and HSV-2 (**D**) infectious viral titers were quantified by plaque assay on Vero cells 24 h posttreatment. Data are shown as means ± SEM, n > 6; results were compared to untreated cultures by ANOVA and post hoc Tukey’s HSD; * *p* < 0.05; ** *p* < 0.01; *** *p* < 0.001.

Neither EPI nor CORT were able to induce HSV-1 reactivation in sensory TG or para-sympathetic CG neurons. However, both EPI and CORT induced HSV-1 reactivation in sympathetic SCG neurons, as shown by significantly increased viral DNA compared to untreated latently infected neurons (UNT; ACV removed but no other treatment) (Figure 1A). Trichostatin A (TSA), a nonspecific histone deacetylase (HDAC) inhibitor that has previously been shown to reactivate HSV-1 reliably from latency [30,33,34], was used as a positive reactivation control. Although EPI and CORT both induced HSV-1 reactivation in sympathetic neurons, viral DNA did not reach the same quantity as TSA-induced reactivation, suggesting that stress hormone-induced reactivation may not be as strong of a stimulus as some other reactivation stimuli. In contrast, CORT induced reactivation of HSV-2 in both sensory TG and sympathetic SCG neurons (Figure 1B), but EPI had no effect on HSV-2, regardless of neuron type. Although we also observed a trend of increased HSV-2 DNA in parasympathetic CG neurons following CORT treatment, neither EPI nor CORT had a statistically significant effect on HSV-1 or HSV-2 in parasympathetic CG neurons. TSA produced a significant increase in HSV-1 and HSV-2 DNA in sensory TG and sympathetic SCG, but not in parasympathetic CG neurons (Figure 1A,B), suggesting that parasympathetic CG neurons may regulate HSV differently than sensory or sympathetic neurons.

To verify that EPI- and CORT-induced reactivation progressed through the complete virus cycle to produce infectious progeny, latently infected neurons were treated with the hormones and infectious viral titers were quantified by plaque assay on Vero cells. Plaque titration results verified that reactivation induced by EPI and CORT progressed through the complete virus cycle; infectious virus titers correlated with increased viral DNA in sympathetic SCG neurons treated with EPI, CORT, and TSA for HSV-1 (Figure 1C) and both TG and SCG neurons treated with CORT and TSA for HSV-2 (Figure 1D). Untreated, latently infected neurons remained negative by plaque assay, demonstrating that these cultures remained latent.

Taken together, our results show that EPI and CORT have differential effects on HSV-1 and HSV-2 reactivation in a neuron-specific manner. EPI induces HSV-1 reactivation only in sympathetic neurons, but has no effect on HSV-2. CORT induces HSV-1 only in sympathetic neurons, but induces HSV-2 reactivation in both sensory and sympathetic neurons.

### 3.2. Adrenergic Receptor Specificity of EPI-Induced Reactivation of HSV-1

EPI can bind to and activate several different adrenergic receptors that are expressed by peripheral neurons, including alpha 1 and 2 (α1-AR, α2-AR) and beta 1 and 2 (β1-AR, β2-AR). Therefore, we next sought to determine which adrenergic receptors are important for the EPI-induced reactivation of HSV-1. We previously demonstrated that adrenergic receptor expression is maintained in cultured primary adult murine neurons similarly to expression in vivo [16]. Furthermore, we showed that HSV-1 antigens generated during productive infection are detected in neurons expressing adrenergic receptors [16].

**Figure 2:**
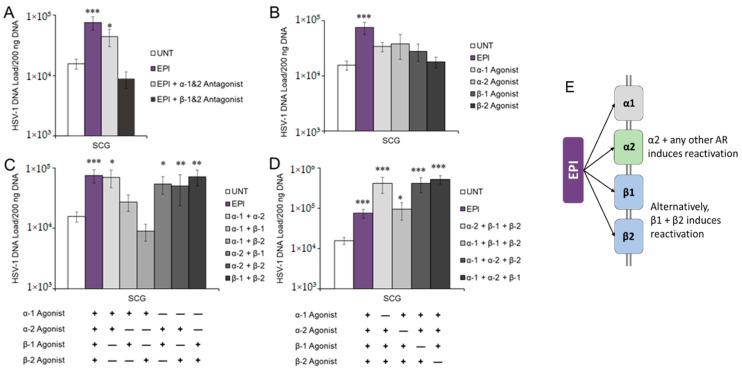
Multiple adrenergic receptors are required for EPI-induced HSV-1 reactivation in sympathetic neurons. (**A**) Reactivation of HSV-1 with EPI in primary adult murine sympathetic SCG cultures either alone, with nonspecific α-AR antagonist (phentolamine), or nonspecific β-AR antagonist (timolol). (**B**) Reactivation of HSV-1 in primary adult murine SCG cultures with either EPI, α-1 agonist (phenylephrine), α-2 agonist (clonidine), β-1 agonist (dobutamine), or β-2 agonist (terbutaline). (**C**) Reactivation of HSV-1 in primary adult murine SCG cultures with either EPI or a combination of two adrenergic agonists utilized in (**B**). (**D**) Reactivation of HSV-1 in primary adult murine SCG cultures with either EPI or a combination of three adrenergic agonists utilized in (**B**). Reactivation was quantified in (**A**–**D**) by viral DNA collected 24 h posttreatment measured by qPCR. (**E**) Data summary: activation of α2 and any other AR induces HSV-1 reactivation in adult sympathetic SCG neurons. Alternatively, activation of β1 and β2 simultaneously can also induce reactivation. Data are means ± SEM, n > 6; results were compared to untreated cultures by ANOVA and post hoc Tukey’s HSD; * *p* < 0.05; ** *p* < 0.01; *** *p* < 0.001.

Since EPI only induced HSV-1 reactivation in sympathetic SCG neurons, we treated latently infected SCG neurons with EPI and nonspecific adrenergic receptor antagonists (blockers) to block activation of either α- or β-ARs. The nonspecific β-AR antagonist (blocker) timolol, which blocks both β1- and β2-AR activation, inhibited EPI-induced HSV-1 reactivation (Figure 2A), suggesting that β-AR activation is necessary to induce HSV-1 reactivation following EPI treatment. In contrast, latently infected neurons treated with the nonspecific α-AR antagonist phentolamine produced quantities of HSV-1 DNA that were still significantly higher than untreated sympathetic neurons (Figure 2A), showing that general blockade of α-ARs cannot effectively block EPI-induced HSV-1 reactivation in sympathetic SCG neurons.

To further assess receptor involvement, we reasoned that if EPI induces reactivation through a specific adrenergic receptor, an agonist that activates that specific receptor would also induce reactivation without EPI treatment. Therefore, SCG neurons latently infected with HSV-1 were treated with agonists for one of four different adrenergic receptors: α1, α2, β1, or β2. In comparison to untreated (UNT) neurons and those treated with EPI, which induced reactivation in those neurons, none of the four adrenergic receptor agonists alone significantly induced reactivation of HSV-1 in sympathetic SCG neurons (Figure 2B).

Since a nonspecific beta blocker was able to prevent EPI-induced HSV-1 reactivation but individual receptor agonists were unable to induce reactivation, we reasoned that activation of a single adrenergic receptor is insufficient for HSV-1 reactivation from latency in sympathetic SCG neurons. However, our results suggested that at least one of the β-adrenergic receptors is necessary for EPI-induced reactivation. To determine whether EPI induces HSV-1 reactivation by activating multiple adrenergic receptors, latently infected SCG neurons were treated with agonists for two of the four different adrenergic receptors simultaneously (Figure 2C). Combined treatments containing the α2-AR agonist clonidine induced reactivation similarly to EPI (Figure 2C). However, a combination of β1- and β2-AR agonists also induced reactivation without clonidine. Therefore, combined activation of the α2-AR and any other adrenergic receptor can induce HSV-1 reactivation in sympathetic SCG neurons to the same extent as EPI, but α2-AR activation alone is not sufficient. Alternatively, combined activation of β1- and β2-AR can also induce HSV-1 reactivation to the same extent as EPI. Although treatment with the combination of α1 and α2 agonists induced reactivation (Figure 2C), the nonspecific α1/α2 antagonist was unable to block EPI-induced reactivation (Figure 2A). Thus, reactivation can proceed through activation of β1- and β2-ARs if the α-ARs are blocked.

To verify these results, latently infected neurons were treated with agonists for three of the four different adrenergic receptors, omitting one. We reasoned that if the omitted one was required for reactivation, reactivation would not occur. All treatments induced HSV-1 reactivation, as demonstrated by significantly increased viral DNA following treatment compared to untreated controls (Figure 2D), but combined treatments that included clonidine (α2-AR agonist) induced HSV-1 reactivation above EPI-induced levels. Thus, activation of α2-AR is necessary for the full reactivation potential of HSV-1 in sympathetic neurons, although activation of β1- and β2-AR simultaneously can compensate.

Our results also suggest that multiple signaling pathways are involved in the process of reactivation, since the adrenergic receptors are coupled to trimeric G-protein complexes that contain different alpha subunits, which activate or inhibit different signaling pathways. The α2-AR couples with the inhibitory G-protein alpha subunit (Gα_i/o_), which inhibits adenylate cyclase and downstream cyclic AMP (cAMP) production, while also activating the Src pathway leading to cAMP response element binding protein (CREB) activation [35]. The α2-AR also couples with the G-protein alpha subunit q (Gα_q_), activating phospholipase C (PLC) and downstream mitogen-activated protein kinase (MAPK) cascade, resulting in activation of CREB and c-Jun terminal kinase (JNK) [36,37]. While β1-AR couples with Gα_s_, which stimulates adenylate cyclase and downstream cAMP production, the β2-AR can couple with either the Gα_s_ or the Gα_i/o_ proteins [38]. Additionally, different Gα proteins modulate β-catenin signaling, with Gα_s_ potentiating and Gα_q_ suppressing the β-catenin-signaling pathway [39]. Thus, EPI activation of several different signaling pathways simultaneously is necessary for the full reactivation of HSV-1.

### 3.3. Corticosteroid Receptor Specificity of CORT-Induced Reactivation of HSV-1 and HSV-2

CORT can bind to and activate glucocorticoid (GR) and mineralocorticoid (MR) receptors [40]. We previously showed that receptor expression is maintained in cultured primary adult murine sensory TG and sympathetic SCG neurons, and that HSV antigens generated during productive infection are present in the same neurons as receptor expression [16]. As shown in Figure 1, CORT induced reactivation of HSV-1, but only in sympathetic SCG neurons, and reactivation of HSV-2 in both sensory TG and sympathetic SCG neurons.

To determine whether CORT-induced reactivation occurs through binding of CORT to GR or MR, latently infected neurons (SCG for HSV-1, SCG and TG for HSV-2) were treated with the GR agonist dexamethasone (DEX) or the MR agonist aldosterone (ALDO).

**Figure 3:**
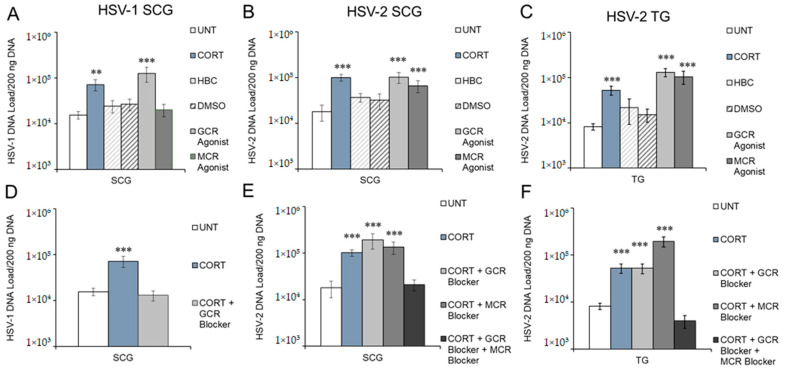
GR is required for CORT-induced HSV-1, but GR and MR are redundant for HSV-2 reactivation. Primary adult neuronal cultures from sensory trigeminal (TG) or sympathetic superior cervical (SCG) ganglia were infected with HSV-1 (**A**,**D**) or HSV-2 (**B**,**C**,**E**,**F**) in the presence of acyclovir for 7 days, followed by treatment with corticosterone (CORT), receptor agonists, or receptor antagonists. (**A**) HSV-1 or (**B**) HSV-2 latently infected sympathetic SCG neuronal cultures were treated with CORT, CORT vehicle HBC, agonist vehicle DMSO, GR agonist (dexamethasone), or MR agonist (aldosterone). (**C**) HSV-2 latently infected sensory TG neuronal cultures were treated with CORT, CORT vehicle HBC, agonist vehicle DMSO, GR agonist (dexamethasone), or MR agonist (aldosterone). (**D**) HSV-1 latently infected SCG neuronal cultures were treated with CORT, either alone or with GR antagonist (mifepristone). HSV-2 latently infected SCG (**E**) or TG (**F**) neuronal cultures were treated with CORT, either alone, with GR antagonist (mifepristone), with MR antagonist (eplerenone), or with both the GR and MR antagonists. Viral DNA was quantified in A–F by DNA collected 24 h posttreatment measured by qPCR. Data are means ± SEM, n > 6; results were compared to untreated cultures by ANOVA and post hoc Tukey’s HSD; ** *p* < 0.01; *** *p* < 0.001.

The GR agonist (DEX), but not the MR agonist (ALDO), induced HSV-1 reactivation similarly to CORT in sympathetic SCG neurons (Figure 3A), demonstrating that HSV-1 reactivates following activation of the GR, but not the MR. HBC (2-hydroxypropyl-β-cyclodextrin), the vehicle for CORT, and DMSO (dimethyl sulfoxide), the vehicle for the CORT agonists and antagonists, had no significant effects on the latent virus (Figure 3A–C). To determine if we could block CORT-induced reactivation of HSV-1 in sympathetic neurons, SCG neurons latently infected with HSV-1 were treated with CORT and the GR antagonist mifepristone, which effectively inhibited CORT-induced reactivation of HSV-1 (Figure 3D). Thus, CORT-induced HSV-1 reactivation occurs through the GR.

In both sympathetic SCG (Figure 3B) and sensory TG neurons (Figure 3C), both GR and MR agonists (DEX and ALDO) individually induced HSV-2 reactivation similar to CORT treatment. Thus, HSV-2 reactivates following activation of either GR or MR in sympathetic and sensory neurons. Neither the GR antagonist mifepristone nor the MR antagonist eplerenone alone was able to block CORT-induced HSV-2 reactivation in SCG neurons (Figure 3E) or TG neurons (Figure 3F). However, administration of mifepristone and eplerenone together, along with CORT, effectively blocked CORT-induced reactivation in both sympathetic SCG and sensory TG neurons (Figure 3E,F). Therefore, CORT is able to induce HSV-2 reactivation in sympathetic SCG and sensory TG neurons through activation of either the GR or MR.

Our results show that CORT induces HSV-1 reactivation only in sympathetic neurons through activation of the GR, while CORT induces HSV-2 reactivation in sensory and sympathetic neurons through either the GR or MR. The GR and MR are cytosolic ligand-activated receptors that can positively or negatively modulate gene expression by binding directly to hormone response elements and recruiting transcription regulatory complexes, or by interacting with other transcription factors. The specificity of GR and MR occurs through binding affinities of the receptors for CORT and ALDO, hormone response element sequences, and posttranslational modifications. Thus, CORT-induced reactivation is selective and the mechanisms likely differ for HSV-1 and HSV-2.

### 3.4. Kinetics of HSV Gene Expression Differ after EPI and CORT Treatment, but Also Depend on Neuron Type

As HSV-1 reactivates in sympathetic SCG neurons using different neuronal receptors in response to EPI and CORT treatment, theoretically, viral gene expression may also differ between EPI and CORT treatments. Kim et al., 2012, categorized HSV reactivation into a biphasic pattern that was time-dependent, in which Phase I transitioned into Phase II at approximately 25 h post-reactivation, using phosphatidyl inositol 3-kinase (PI3K) inhibitor LY294002 as a reactivation stimulus. As our focus was on early events post-stimulus, we quantified the HSV-1 immediate early (IE) gene expression of ICP0, ICP4, and ICP27 during latency (0 h timepoint, prior to hormone treatment) and between 1 and 25 h after EPI and CORT treatment, using droplet digital PCR (ddPCR) to evaluate the expression profiles of these IE genes in primary adult neurons. In addition, we also quantified viral protein 16 (VP16) transcript expression over the same time-course, as previous studies have reported the involvement of trans-activator VP16 in facilitating the transition from latency to a productive infection following a stress-induced stimulus [41,42].

**Figure 4:**
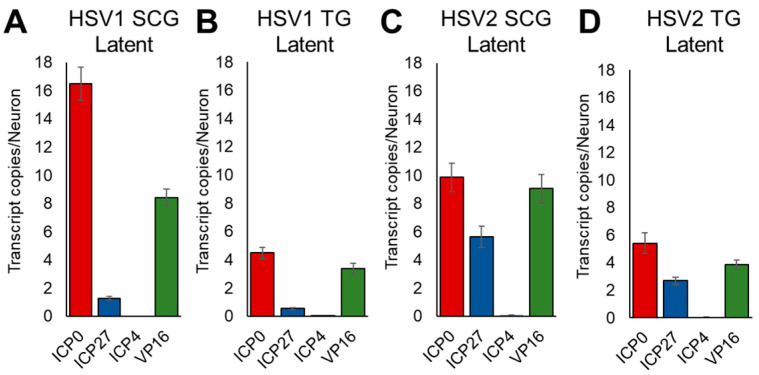
HSV-1 and HSV-2 ICP0, ICP27, ICP4, and VP16 gene expression in SCG and TG neurons during latency. Gene expression was quantified by droplet digital PCR (ddPCR) 7 days after infection in the presence of acyclovir (300 μM). Data is shown as gene transcripts per neuron. (**A**) HSV-1 in SCG neuronal cultures. (**B**) HSV-1 in TG neuronal cultures. (**C**) HSV-2 in SCG neuronal cultures. (**D**) HSV-2 in TG neuronal cultures. Data are means ± SEM, n > 6.

During latency, we detected low levels of expression of ICP0, ICP27, and VP16 in both HSV-1- and HSV-2-infected sensory and sympathetic neurons, shown as number of transcripts per neuron (Figure 4). Although expression level varied depending upon HSV type and the neurons in which the viruses established latency, the general pattern of expression was similar (Figure 4). ICP0 was the most highly expressed (4.5–16.5 transcripts/neuron), followed by VP16 (3.4–9.1 transcripts/neuron). ICP27 was more highly expressed by HSV-2 (2.7–5.6 transcripts/neuron; Figure 4C,D) than HSV-1 (0.5–1.3 copies/neuron; Figure 4A,B). In contrast, ICP4 showed negligible expression in both TG and SCG neurons during latency (Figure 4A–D). For comparison and to demonstrate latency, we also detected up to 70.65 copies of the latency-associated transcript (LAT) per neuron.

**Figure 5:**
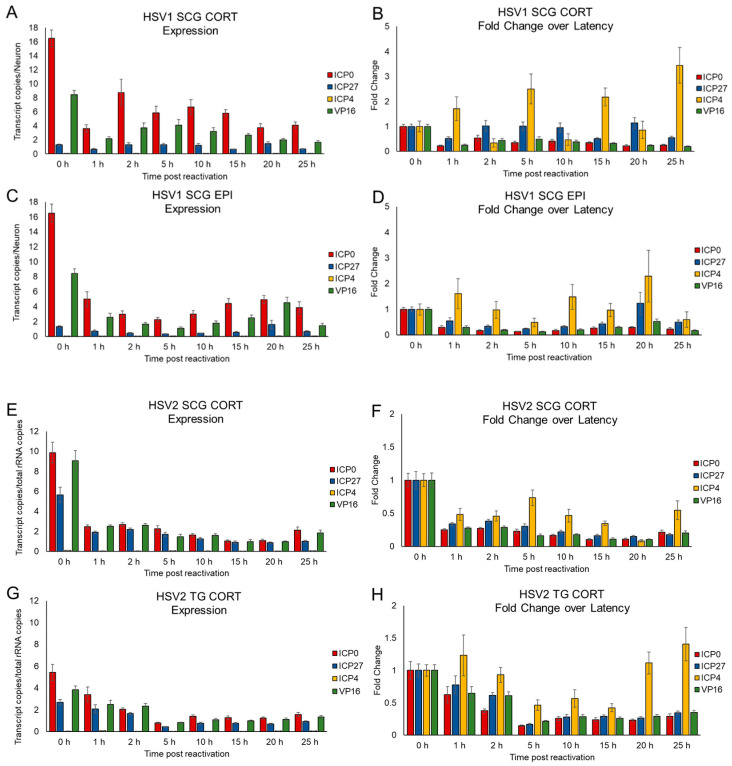
HSV gene expression in primary SCG and TG neuronal cultures during EPI- and CORT-induced reactivation. Primary adult neuronal cultures from sympathetic SCG and sensory TG infected with HSV-1 or HSV-2 for 7 days in the presence of acyclovir to establish latency were treated with epinephrine (EPI) or corticosterone (CORT) to induce reactivation. Total RNA was extracted at the designated timepoints post-reactivation, and viral gene transcripts for ICP0, ICP27, ICP4, and VP16 were quantified by ddPCR. (**A**) HSV-1 in SCG treated with CORT, presented as gene transcript copies per neuron. (**B**) HSV-1 in SCG treated with CORT, presented as fold-change compared to each gene expressed during latency with no treatment and no removal of acyclovir (0 h). (**C**) HSV-1 in SCG treated with EPI, as gene transcripts per neuron. (**D**) HSV-1 in SCG treated with EPI, as fold-change compared to each gene expressed during latency (0 h). (**E**) HSV-2 in SCG treated with CORT, as gene transcript copies per neuron. (**F**) HSV-2 in SCG treated with CORT, as fold-change compared to each gene expressed during latency (0 h). (**G**) HSV-2 in TG treated with CORT, as gene transcripts per neuron. (**H**) HSV-2 in TG treated with CORT, as fold-change compared to each gene expressed during latency (0 h). Data are means ± SEM, n > 6 experiments.

Following treatment with EPI and CORT, we show gene expression as both transcript copies per neuron, as well as fold-change over latent data (Figure 5), as neither alone provides a complete picture of expression kinetics. HSV-1 reactivates in sympathetic SCG neurons in response to either CORT or EPI treatment (see Figure 1). In SCG neurons latently infected with HSV-1, CORT treatment reduced expression of ICP0 and VP16, but ICP27 expression remained stable over 25 h (Figure 5A). Although the change in ICP4 expression cannot be discerned in Figure 5A (transcript copies per neuron), Figure 5B (fold-change in expression) better illustrates the significant increase in ICP4 expression following CORT treatment compared with expression levels during latency. Following EPI treatment, gene expression was also reduced for ICP0 and VP16, although expression increased between 10 h and 20 h posttreatment (Figure 5C). Similarly, ICP4 expression increased almost immediately following treatment, fell to below-latency levels, and then increased again between 10 and 20 h (Figure 5D). ICP27 increased transiently at 20 h posttreatment, but otherwise, expression remained relatively stable throughout the 25 h time period.

HSV-2 reactivates in both sensory TG and sympathetic SCG neurons in response to only CORT treatment (see Figure 1) through either the GR or the MR (see Figure 3). Our lab has already established that GR expression differs between sensory TG neurons and sympathetic SCG neurons [16]. Following CORT treatment in SCG or TG neurons, expression of ICP0, VP16, and ICP27 decreased within 1 h compared to latency, and expression remained relatively constant over the next 25 h (Figure 5E,G). ICP4 expression also remained low in SCG neurons, below the level of expression during latency (Figure 5F), but fluctuated in TG neurons, increasing above latency levels at 25 h posttreatment (Figure 5H).

Overall, the temporal profiles of IE gene and VP16 expression varied by virus and neuron type posttreatment with EPI and CORT. Taken together, our data suggest HSV-1 and HSV-2 gene expression during the initial phase of reactivation, immediately following the stimulus, differs depending on the reactivation stimulus and the type of neuron in which the virus is latent.

### 3.5. Inhibition of Protein Kinases and Transcription Factors Selectively Block Stress Hormone-Induced Reactivation of HSV-1 and HSV-2

As G-protein-coupled receptors, adrenergic receptors (α1, α2, β1, β2) are linked to different G-protein alpha (Gα) subunits. Thus, EPI binding to these adrenergic receptors can activate Gα_s_, Gα_i/o_, or Gα_q_ proteins, which, in turn, can stimulate or inhibit adenylate cyclase and subsequent activation of cAMP or activate phospholipase C (PLC) to activate the mitogen-activated protein kinase (MAPK) pathway. Ultimately, these signaling pathways either activate or inhibit protein kinases and transcription factors, such as cAMP response element binding protein (CREB), c-Jun N-terminal kinase (JNK), protein kinase B (Akt), and nuclear factor kappa-light-chain-enhancer of activated B cells (NFκB) (see a simplified signaling diagram in Figure 7). Upon activation by CORT, the GR and MR transport into the nucleus to directly bind the DNA of target genes, recruiting the p300/CBP coactivator complex to activate gene transcription through histone acetylation [43]. However, the GR also regulates other signaling pathways that include CREB, JNK, and β-catenin (see Figure 7). Previously, JNK- and CREB-responsive elements were shown to be important in stress-induced reactivation of HSV-1 [44,45,46]. The transcription factor β-catenin plays a role in bovine herpesvirus 1 (BoHV-1) latency and reactivation [47,48], and induction of β-catenin expression decreased HSV-1 replication in vitro, suggesting a possible role in the establishment and maintenance of latency [49]. Therefore, we sought to determine if CREB, JNK, and β-catenin are important in adrenergic and glucocorticoid-induced reactivation of HSV-1 and HSV-2.

**Figure 6:**
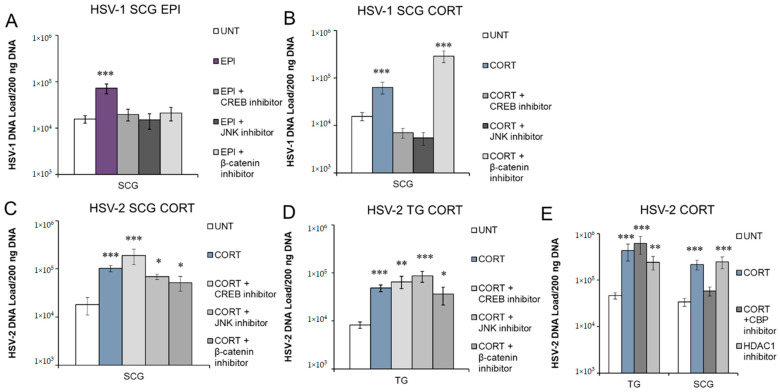
Inhibition of transcription factors and protein kinases differentially affect EPI- and CORT-induced reactivation. Primary adult neuronal cultures from sympathetic SCG or sensory TG were infected with HSV-1 or HSV-2 in the presence of acyclovir for 7 days to establish latency, prior to treatment with EPI or CORT and inhibitors. HSV-1 in SCG neuronal cultures treated with EPI (**A**) or CORT (**B**), either alone or with inhibitors for CREB (666-15), JNK (SP600125), or β-catenin (iCRT14). HSV-2 in SCG (**C**) or TG (**D**) cultures treated with CORT either alone or with CREB, JNK, or β-catenin inhibitors. (**E**) HSV-2 in SCG and TG cultures treated with CORT, CORT with p300/CBP inhibitor C646, or HDAC1 inhibitor pyroxamide alone. Viral DNA was quantified in A–E by viral DNA collected 72 h posttreatment, measured by qPCR. Data are means ± SEM, n > 3; * *p* < 0.05; ** *p* < 0.01; *** *p* < 0.001.

To determine if we could prevent EPI- or CORT-induced reactivation by inhibiting activation of CREB, latently infected neurons were treated with EPI or CORT and the CREB inhibitor 666–15. This inhibitor prevents the phosphorylation of CREB at serine 133 by various cellular serine/threonine protein kinases, such as protein kinase A (PKA) or mitogen-activated protein kinases (MAPKs). Phosphorylation allows CREB to interact with CREB-binding protein (CBP) and p300, which are essential for CREB-mediated gene transcription [50]. Inhibition of CREB activity blocked both EPI- and CORT-induced reactivation of HSV-1 in sympathetic SCG neurons (Figure 6A,B). In contrast, inhibition of CREB did not prevent CORT-induced reactivation of HSV-2 in either sympathetic SCG (Figure 6C) or sensory TG (Figure 6D) neurons. Thus, CREB activation is an essential event during stress hormone-induced HSV-1 reactivation, but not for HSV-2.

SP600125, which competitively inhibits ATP binding to JNK, thus reducing JNK’s ability to phosphorylate c-Jun [51], was previously shown to effectively inhibit HSV-1 reactivation induced by nerve growth factor (NGF) deprivation in embryonic sympathetic SCG neuronal cultures [46]. In latently infected adult SCG neurons, the JNK inhibitor SP600125 effectively prevented both EPI- and CORT-induced reactivation of HSV-1, as expected (Figure 6A,B). In contrast, inhibition of JNK was unable to prevent CORT-induced reactivation of HSV-2 in either sympathetic SCG (Figure 6C) or sensory TG (Figure 6D) neurons. Thus, JNK activity is an essential component of HSV-1 reactivation in response to several different reactivation stimuli, including EPI and CORT treatment in our studies and NGF deprivation in previous studies [46], but it does not appear to be essential for HSV-2 reactivation in either sympathetic or sensory neurons. Alternatively, another signaling cascade may serve as a redundant pathway if JNK is inhibited during HSV-2 reactivation.

Under resting conditions, cytoplasmic β-catenin is bound to its destruction complex, which results in its ubiquitination and degradation to maintain a basal level of β-catenin in the cell [52]. In canonical Wnt signaling, β-catenin is released from the destruction complex, enters the nucleus, and binds T cell factor (TCF) to activate the transcription of target genes [52]. A β-catenin inhibitor, iCRT14, inhibits the interaction between nuclear β-catenin and the N-terminal domain of TCF to block the transcriptional targets of β-catenin signaling [53]. In our studies, administration of iCRT14 prevented adrenergic reactivation of HSV-1 in sympathetic SCG neurons (Figure 6A). Thus, β-catenin interaction with TCF in the nucleus appears to play a role in adrenergic reactivation of HSV-1 in sympathetic neurons, likely through activation of specific viral or cellular target genes.

In contrast, β-catenin inhibition did not prevent CORT-induced reactivation of HSV-1 (Figure 6B), nor did it block HSV-2 reactivation in either sympathetic SCG or sensory TG neurons (Figure 6C,D). These results suggest that the mechanism of glucocorticoid-induced reactivation is not dependent on β-catenin–TCF activation of gene transcription. Since activated GR also binds directly to DNA and recruits CBP/p300 to acetylate histones, we also used a p300/CBP inhibitor (C646) to determine if we could prevent CORT-induced HSV-2 reactivation by inhibition of the histone acetyltransferase (HAT) activity of p300/CBP. Although CORT-induced reactivation was not prevented in sensory TG neurons, inhibition of p300/CBP HAT activity effectively prevented CORT-induced reactivation of HSV-2 in sympathetic SCG neurons (Figure 6E). Since histone deacetylase 1 (HDAC1) and p300 can directly bind to overlapping regions of the histone H3 tail and compete for histone binding [54], we attempted to induce reactivation of HSV-2 with an HDAC1-specific inhibitor, pyroxamide. In sympathetic SCG neurons, pyroxamide alone induced HSV-2 reactivation similarly to CORT-induced reactivation (Figure 6E). Thus, recruitment of p300/CBP and its associated HAT activity are required for CORT-induced reactivation of HSV-2 in adult sympathetic neurons, while deacetylation by HDAC1 assists in the maintenance of the latent state. Although we speculate that chromatin regulation is also the primary regulator for HSV-2 in sensory TG neurons, further studies are needed to determine the precise signaling pathway that induces the necessary changes for HSV-2 reactivation to occur in sensory TG neurons. While CORT induces HSV-2 reactivation in sensory TG neurons, we were unable to identify a specific inhibitor that could effectively block CORT-induced reactivation in these neurons, demonstrating that multiple signaling pathways can induce reactivation of HSV-2. Further studies are also needed to clarify the role of β-catenin in reactivation, since this factor appears to be a key mediator in differentiating cellular signaling events activated by EPI and CORT.

## 4. Discussion

In our studies, we demonstrate that the stress hormones epinephrine (EPI) and corticosterone (CORT) induce reactivation of HSV-1 selectively in sympathetic, but not in sensory or parasympathetic primary adult neurons. We also demonstrate that activation of more than one adrenergic receptor is necessary for EPI to induce HSV-1 reactivation, while only the glucocorticoid receptor is required for CORT-induced reactivation of HSV-1. In contrast, CORT induces HSV-2 reactivation in both sensory and sympathetic neurons, but EPI has no significant effects on HSV-2 latent infection. Furthermore, CORT can induce HSV-2 reactivation through either the glucocorticoid or mineralocorticoid receptors. The temporal kinetics of ICP0, ICP27, ICP4, and VP16 indicate that different reactivation stimuli induce neuron-specific patterns of viral gene expression. Thus, our studies demonstrate that reactivation mechanisms for HSV are neuron-specific, stimulus-specific, and virus-specific.

### 4.1. HSV-1

Previously, we demonstrated that EPI treatment during productive infection enhanced HSV-1 DNA replication and infectious virus titer production in sympathetic, but not sensory, primary adult neurons [16]. Our current studies correlate with these findings, demonstrating that EPI also induces reactivation of HSV-1 in the same neurons. However, CORT decreased HSV-1 DNA replication and infectious virus titer during productive infection [16], while our current studies show that CORT induces reactivation from latency. Previous research has demonstrated that glucocorticoid treatment during productive infection can reduce the expression of both viral lytic genes and host inflammatory genes, and that the activated GR can bind to one of the origins of replication in the HSV-1 genome, potentially limiting viral gene expression, genome replication, and severity of acute disease [55,56].

During reactivation from latency, the viral genome exists under epigenetic repression and must respond to a reactivation stimulus. Kim et al. (2012) categorized HSV-1 reactivation into a biphasic pattern, in which Phase I served as the priming phase 15–24 h post-reactivation (hpr), characterized by the dysregulated expression of all classes of lytic transcripts, while Phase II (from ~25 hpr onward) resulted in genome amplification and viral progeny similar to productive infection. Their studies utilizing embryonic sympathetic SCG neurons and PI3-K inhibition as a reactivation stimulus demonstrated that ICP27 and VP16 were expressed at 20 hpr and decreased at 25 hpr. We observed a similar pattern in HSV-1 latently infected adult sympathetic SCG neurons treated with EPI, although we observed a greater increase in ICP4 than VP16 at 20 hpr compared to expression levels during latency. Our data show that EPI and CORT induced gene expression for ICP0, ICP27, ICP4 and VP16 with different temporal kinetics. However, both treatments resulted in viral reactivation from latency. The contrasting viral transcript expression kinetics between EPI and CORT treatment suggest that these two reactivation stimuli activate different signaling mechanisms that result in HSV-1 reactivation in sympathetic neurons. Our inhibition studies support this finding as well, demonstrating that inhibiting β-catenin interaction with TCF can prevent EPI-induced reactivation of HSV-1 but not CORT-induced reactivation. Although CREB and JNK are common factors downstream of both EPI and CORT, adrenergic and glucocorticoid receptor stimulation clearly result in the activation of different signaling cascades.

**Figure 7:**
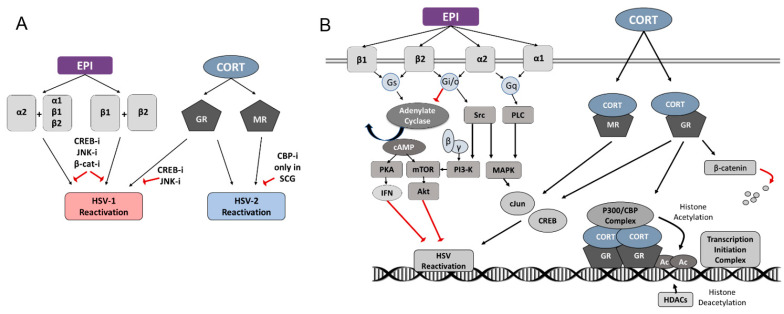
Adrenergic and glucocorticoid signaling pathways and HSV reactivation pathways. (**A**) Epinephrine (EPI) induces HSV-1 reactivation through the activation of α2 and any other AR, or through the activation of β1 and β2, which can be blocked by the inhibitors of CREB, JNK, or β-catenin. Corticosterone (CORT) induces HSV-1 reactivation through the glucocorticoid receptor (GR), which can be blocked by the inhibitors of CREB or JNK. CORT induces HSV-2 reactivation through either glucocorticoid (GR) or mineralocorticoid (MR) receptors; the only factor we identified able to block HSV-2 reactivation was a CBP inhibitor, and only in SCG neurons. (**B**) Epinephrine (EPI) activates multiple adrenergic receptors on peripheral neurons (α1, α2, β1, β2), which couple with different G-protein α subunits (Gq, Gi/o, Gs), which can activate different signaling cascades. Corticosterone (CORT) activates signaling cascades through cytosolic glucocorticoid (GR) and mineralocorticoid (MR) receptors. Cytosolic effector proteins and transcription factors mediated by EPI and CORT signaling include adenylate cyclase, cyclic AMP (cAMP), protein kinase A (PKA), interferons (IFN), the mechanistic target of rapamycin (mTOR), protein kinase B (Akt), protein kinase Src, phosphoinositide 3-kinase (PI3-K), phospholipase C (PLC), mitogen-activated protein kinases (MAPKs), c-Jun, cAMP response element-binding protein (CREB), CREB binding protein (CBP), p300/CBP Complex, histone deacetylases (HDACs), and β-catenin. Black arrows indicate stimulation or activation; red arrows indicate inhibition.

We also show that EPI must activate multiple adrenergic receptors to induce the reactivation of HSV-1 from sympathetic neurons (Figure 7A). Since the ARs couple with different G-protein α subunits, EPI can activate or inhibit adenylate cyclase and downstream cyclic AMP (cAMP) production, while also activating Src [35] or phospholipase C (PLC) pathways (Figure 7B) [36,38]. Additionally, different Gα proteins modulate β-catenin signaling, with Gα_s_ potentiating and Gα_q_ suppressing β-catenin signaling [39]. The common denominators in the signaling pathways that induce adrenergic reactivation are the activation of CREB and JNK, modulation of β-catenin, and Gα_i/o_ inhibition of the adenylate cyclase pathway, which, in turn, inhibits downstream factors NFκB and Akt. In our studies, CREB, JNK, and β-catenin inhibition blocked EPI-induced reactivation, demonstrating that each of these factors can independently play a role in the reactivation process. However, a complex network of signaling events must occur simultaneously for EPI to induce HSV-1 reactivation in adult sympathetic neurons.

Stimulation of the glucocorticoid receptor (GR) by CORT also induced HSV-1 reactivation in sympathetic neurons, but not sensory neurons, although the GR is expressed in both sensory TG [15] and sympathetic SCG neurons [16]. In sensory neurons, activation of either the GR or the ARs was not sufficient to activate or disrupt the necessary signaling pathways to reach the threshold required for the reactivation of HSV-1. Iontophoresis of epinephrine or administration of the GR agonist dexamethasone (DEX) have been shown to reactivate HSV-1 in the rabbit and mouse models of ocular infection [11,12,57,58,59], although our results suggest that reactivation may have occurred in sympathetic SCG rather than sensory TG neurons in these animal models. DEX can stimulate the expression of a reporter gene from the HSV-1 ICP0 promoter by activating several host transcription factors [22,24]. In cooperation with Krüppel-like transcription factors (KLF15 and KLF4), the GR has also been shown to transactivate ICP27 and ICP4 [25,26], which are the viral genes that increased early in response to CORT treatment in our studies. Both JNK and CREB activity were required for CORT-induced HSV-1 reactivation in sympathetic SCGs, but in contrast to EPI-induced HSV-1 reactivation, β-catenin did not appear to be involved.

Reactivation of HSV-1 from sympathetic neurons depends upon downstream transcription factors and protein kinases. While adrenergic reactivation requires the stimulation of multiple adrenergic receptors, CORT-induced reactivation only required activation of the GR, not the MR (Figure 7A). Reactivation from both stress hormone pathways was blocked when CREB and JNK were inhibited, suggesting that there are commonalities between the mechanisms of reactivation by these stress hormones. However, the pathways are not necessarily equivalent, considering that β-catenin is only implicated in adrenergic reactivation, and the kinetics of IE and VP16 gene expression differed between the two stress hormones. Considering that neither EPI nor CORT induced HSV-1 reactivation above untreated levels in primary adult sensory neurons, and we have previously demonstrated that HSV-1 reactivates in these neurons in response to the deprivation of the neurotrophic factor neurturin (NTN) [32], we conclude that different mechanisms are responsible for HSV-1 reactivation, depending on the type of neuron in which the latent virus resides and the nature of the reactivation stimulus.

### 4.2. HSV-2

In contrast to HSV-1, EPI does not induce HSV-2 reactivation, regardless of neuron type. Furthermore, the GR and the MR are redundant pathways for CORT-induced reactivation, with activation of either receptor being sufficient to induce HSV-2 reactivation. However, inhibition of the potential downstream effectors of GR and MR that we tested, including CREB, β-catenin, and JNK, did not block HSV-2 reactivation in either sympathetic SCG or sensory TG neurons. These results suggest that the mechanism by which CORT induces HSV-2 reactivation likely involves either the chromatin remodeling properties of the GR or GR’s ability to recruit transcription factors that activate genes needed for reactivation. Alternatively, multiple signaling pathways are responsible for HSV-2 reactivation and a combinatorial blockade would be necessary to prevent it.

Our ability to block CORT-induced HSV-2 reactivation with a p300/CBP histone acetylase (HAT) inhibitor supports a mechanism regulated by histone modifications in sympathetic SCG neurons. Activated GR transports into the nucleus to directly bind the DNA of target genes, recruiting the p300/CBP initiation complex, which acetylates histones and initiates gene transcription [60]. The histone acetylation of immediate early (IE) gene promoters has been associated with HSV-1 reactivation [61,62]. CBP and p300 are recruited to ICP4, ICP0, and ICP27 promoters during HSV-1 productive infection [63], suggesting that activated GR could potentially induce reactivation of HSV-2 through the histone acetylation of IE gene promoters.

We found changes in HSV-2 gene expression of ICP4, ICP0, ICP27, and VP16 after CORT treatment in both sensory and sympathetic neurons, although the specific pattern of gene expression varied by neuron type. However, the kinetic profile of these IE genes and VP16 did not increase beyond untreated levels in HSV-2-infected neurons in the first 25 h posttreatment, although ICP4 expression was trending upwards. These results suggest that HSV-2 latency is not a completely dormant state, but a dynamic process that maintains a basal level of viral gene expression, which maintains the neuronal environment and the viral genome in a ready-state to respond to a reactivation stimulus. In our studies, ICP0 was expressed at low levels during latency; as an E3 ubiquitin ligase, basal expression of ICP0 during latency could potentially target specific neuronal proteins for degradation to maintain the latent state. Upon a reactivation stimulus, ICP0 expression decreased, suggesting that, in the event of a neuronal stressor, the loss of ICP0 may permit the expression of neuronal proteins required for productive infection within the neuron.

Trichostatin A (TSA), a histone deacetylase (HDAC) inhibitor that targets HDACs 1, 3, 4, 6, and 10, has previously been used to induce HSV-1 reactivation in quiescently infected rat pheochromocytoma cells (PC-12) and primary adult sensory and sympathetic neurons [30,33,64]. In our studies, TSA induced HSV-2 reactivation in sensory and sympathetic neurons, but not in parasympathetic neurons. We previously showed that, following ocular infection in guinea pigs, HSV-2 was unable to fully establish latency in parasympathetic ciliary ganglia, either in vivo or in vitro, and instead maintained a persistent lytic infection [65]. The CBP inhibitor was able to prevent CORT-induced reactivation in sympathetic SCG neurons but not in sensory TG neurons. Our findings support the hypothesis that different types of neurons may maintain the HSV-2 viral genome in different histone configurations under the control of different histone modifiers, although the latent state may not be fully suppressed and as well-controlled by some neurons compared to others, or compared to HSV-1. A less repressed viral genome, responsive to multiple reactivation stimuli in both sensory and autonomic neurons, would lead to more frequent clinical recurrences and episodes of viral shedding, as occurs in humans infected with HSV-2.

Both glucocorticoids and epinephrine are crucial hormones required to maintain homeostasis of the body’s key physiological functions. CORT, being a homeostatic anticipatory hormone, is produced on activation of the hypothalamic–pituitary–adrenal (HPA) axis to regulate neuronal and hormonal systems, respond to stress, and maintain central and peripheral circadian rhythm. Traumatic physiological and emotional distress can also activate the adrenal medulla, inducing the secretion of epinephrine, which then acts as a neurotransmitter for the sympathetic system to restore homeostasis [66,67]. The opposing activity of natural CORT and EPI maintains homeostatic balance, but also causes adverse pathophysiological conditions when that balance is disrupted. Thus, EPI and CORT, from a broader physiological perspective, might not elicit a harsh stimulus for reactivation compared to more acute and direct stressors, such as sunburn, UV radiation, or nerve trauma, which is reflected in our results, showing a relatively low level of reactivation. Nonetheless, the hormones did indeed selectively induce reactivation, as exemplified by the production of viral progeny following treatment.

## 5. Conclusions

Stress hormones have differential effects on HSV-1 and HSV-2, demonstrating that the mechanism of reactivation is not the same for the two viruses. Therefore, clinical interventions developed to treat HSV-1 in humans may not work as effectively for HSV-2. Our studies also show that the mechanism of reactivation is not the same between different types of neurons, as exemplified by stress hormone-induced HSV-1 reactivation occurring in sympathetic neurons, but not sensory neurons. Therefore, characteristics of HSV reactivation cannot be generalized to all types of neurons in which HSV-1 and HSV-2 may establish latency. Similarly, mechanisms regulating HSV-1 cannot be generalized to HSV-2. Furthermore, latently infected sympathetic neurons represent an important reservoir of reactivating HSV, particularly in response to hormone stimuli, contributing to differences in recurrence characteristics of HSV-1 and HSV-2. Taken together, reactivation mechanisms for HSV are neuron-specific, stimulus-specific, and virus-specific.

## Data Availability

Further information and requests for resources and reagents should be directed to and will be fulfilled by the lead contact, Andrea S. Bertke (asbertke@vt.edu).
